# Acute Myocardial Infarction Caused by Filgrastim: A Case Report

**DOI:** 10.1155/2012/784128

**Published:** 2012-12-23

**Authors:** Cemil Bilir, Hüseyin Engin, Yasemin Bakkal Temi, Bilal Toka, Turgut Karabağ

**Affiliations:** ^1^Department of Internal Medicine, School of Medicine, Bulent Ecevit University, Zonguldak, Turkey; ^2^Department of Cardiology, School of Medicine, Bulent Ecevit University, Zonguldak, Turkey

## Abstract

Common uses of the granulocyte-colony stimulating factors in the clinical practice raise the concern about side effects of these agents. We presented a case report about an acute myocardial infarction with non-ST segment elevation during filgrastim administration. A 73-year-old man had squamous cell carcinoma of larynx with lung metastasis treated with the chemotherapy. Second day after the filgrastim, patient had a chest discomfort. An ECG was performed and showed an ST segment depression and negative T waves on inferior derivations. A coronary angiography had showed a critical lesion in right coronary arteria. This is the first study thats revealed that G-CSF can cause acute myocardial infarction in cancer patients without history of cardiac disease. Patients with chest discomfort and pain who are on treatment with G-CSF or GM-CSF must alert the physicians for acute coronary events.

## 1. Introduction

Granulocyte-colony stimulating factors (G-CSF) are commonly used in patients with chemotherapy-induced neutropenia. Recently G-CSF has been used in clinical trials to research neovascularization and/or to reduce the damaged size of infarct. Common uses of the granulocyte-colony stimulating factors in the clinical practice raise the concern about side effects of these agents. Studies showed that nearly 5% of patients undergoing peripheral blood stem cell mobilization with G-CSF developed venous thromboembolic events (VTEs) [1]. In addition an early dose escalation study for G-CSF revealed that 5/39 patients had chest pain and 1/39 patient had abnormal ST segment depression [2]. We also presented a case about an acute myocardial infarction with non-ST segment elevation during filgrastim administration. 

## 2. Case Report

**Figure 1:**
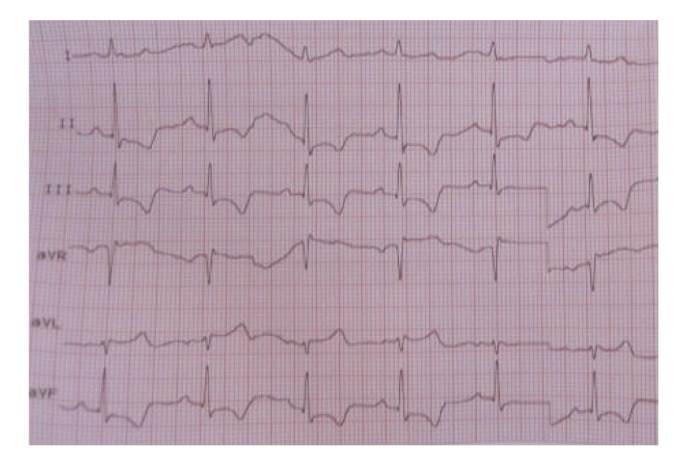
Negative T wave and ST segment depression on inferior derivations.

**Figure 2:**
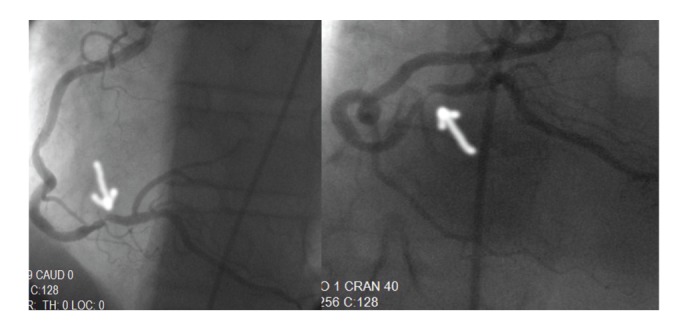
Critical lesion in right coronary arteria.

A 73-year-old man had squamous cell carcinoma of larynx with lung metastasis treated with the chemotherapy including the docetaxel, cisplatin, and fluorouracil regimen. The patient was admitted to the hospital for pneumonia after the 3rd course of chemotherapy. Piperacillin tazobactam of 4∗2.25 gr per day was given. On the 4th day of the treatment of antibiotic, patients become neutropenic without fever, and then filgrastim 5 mcg/kg/day was administered. Patient had a chest discomfort on the second day of filgrastim administration. An ECG was performed, and ST segment depression with negative T waves on inferior derivations of the ECG had been determined (Figure 1). Patient's ECG was normal on admission to the hospital. Cardiac enzymes analysis were elevated, and value of troponin I was 1,9 ng/mL and value of CK-MB was 7,3(5,5) ng/mL. Filgrastim was discontinued on 3rd day, and then cardiac enzymes were normalized on 10th day of the treatment of anticoagulation. Also a coronary angiography was performed, and it showed a critical lesion in right coronary arteria (Figure 2). 

## 3. Discussion

There have been many case reports about the thrombotic events in cancer patients receiving chemotherapy. Most of the case reports were associated with granulocyte-macrophage colony stimulating factors (GM-CSF), but there are no clear data in the literature about G-CSF. We presented a myocardial infarction case during the G-CSF treatment in a patient without history of coronary heart disease. G-CSF and GM-CSF have cardiovascular adverse events. Tolcher et al. had reported 2 cases about the iliac arterial thrombosis related to the GM-CSF, and Waldecker-Herrmann et al. also reported a catheter-related thrombosis of the internal jugular and subclavian vein during GM-CSF [3, 4]. Eckman et al. reported 2 cases, one of them was intraplaque hemorrhage and the other was non-ST-elevated myocardial infarction related to the G-CSF [5]. These patients had a history of coronary heart disease, but our patient did not have a coronary heart disease history. Possible mechanism of the thrombosis include, increased tissue factor expression on macrophages and adhesion molecules on neutrophils. G-CSF increases the CRP levels in healthy subjects, and CRP can stimulate proinflammatory mediators so it can impact on platelet aggregation [6]. A study revealed that, G-CSF induce a hypercoagulable state by increase the levels of FVIII:C and thrombin generation [7]. 

This is the first case report that revealed that G-CSF can cause acute myocardial infarction in cancer patients without a history of cardiac disease. In cancer patients, physicians must alert for acute coronary events when a patient has chest discomfort or chest pain especially during colony stimulating agent treatment.
